# A Tobacco Syringe Agroinfiltration-Based Method for a Phytohormone Transporter Activity Assay Using Endogenous Substrates

**DOI:** 10.3389/fpls.2021.660966

**Published:** 2021-04-06

**Authors:** Jiangzhe Zhao, Min Ju, Jiayun Qian, Mengyuan Zhang, Ting Liu, Kewei Zhang

**Affiliations:** Institute of Plant Genetics and Developmental Biology, Zhejiang Provincial Key Laboratory of Biotechnology on Specialty Economic Plants, Zhejiang Normal University, Jinhua, China

**Keywords:** phytohormone, transporter, transporter activity assay, tobacco syringe agroinfiltration, endogenous substrates

## Abstract

Phytohormones are a group of small chemical molecules that play vital roles in plant development, metabolism, and stress responses. Phytohormones often have distinct biosynthesis and signaling perception sites, requiring long- or short-distance transportation. Unlike biosynthesis and signal transduction, phytohormone transport across cells and organs is poorly understood. The transporter activity assay is a bottleneck for the functional characterization of novel phytohormone transporters. In the present study, we report a tobacco syringe agroinfiltration and liquid chromatography tandem mass spectrometry (TSAL)-based method for performing a phytohormone transporter activity assay using endogenous hormones present in tobacco (*Nicotiana benthamiana*) leaves. A transporter activity assay using this method does not require isotope-labeled substrates and can be conveniently performed for screening multiple substrates by using endogenous hormones in tobacco leaves. The transporter activities of three known hormone transporters, namely AtABCG25 for abscisic acid, AtABCG16 for jasmonic acid, and AtPUP14 for cytokinin, were all successfully validated using this method. Using this method, cytokinins were found to be the preferred substrates of an unknown maize (*Zea mays*) transporter ZmABCG43. ZmABCG43 transporter activities toward cytokinins were confirmed in a cytokinin long-distance transport mutant *atabcg14* through gene complementation. Thus, the TSAL method has the potential to be used for basic substrate characterization of novel phytohormone transporters or for the screening of novel transporters for a specific phytohormone on a large scale.

## Introduction

Phytohormones, including indole-3-acetic acid (IAA), cytokinin (CK), abscisic acid (ABA), brassinosteroids (BR), ethylene (ET), gibberellin (GA), jasmonic acid (JA), salicylic acid (SA), and strigolactone (SL) (Ubeda-Tomás et al., 2012; Al-Babili and Bouwmeester, 2015; Ding and Ding, 2020), are endogenous chemical molecules that play a crucial role in plant growth, development, and response to biotic and abiotic stress (Zhao, 2010; Vanstraelen and Benková, 2012; Yu et al., 2015; Chanclud and Lacombe, 2017). Studies on hormone biosynthesis, metabolism, and signaling have focused on understanding the functional mechanisms and cross-talks between different members (Peleg and Blumwald, 2011). The sites of hormone synthesis sometimes differ from the sites of the action of hormones (Park et al., 2017). Therefore, hormone transport is required for the proper distribution of hormones for signaling.

Transporters are required in the long-distance, short-distance, and cellular transport of phytohormones, through which plants tightly regulate the spatial and temporal distribution of hormones (Fonseca et al., 2014). Characterization of multiple auxin transporters has led to the elucidation of auxin transport mechanisms (Park et al., 2017). PIN-formed transporters, PIN-LIKES (PILS), and ATP-binding cassette (ABC) family proteins have been shown to be involved in auxin transport (Terasaka et al., 2005; Bouchard et al., 2006; Petrasek et al., 2006; Kamimoto et al., 2012; Zhang et al., 2018). Transporters of GA, ABA, SL, SA precursor, and CK have been identified from families, such as the nitrate transporter1/peptide transporter family (NPF) (Saito et al., 2015; Tal et al., 2016), multidrug and toxic compound extrusion (MATE) (Serrano et al., 2013; Zhang H. et al., 2014; Rekhter et al., 2019), ABC (Kuromori et al., 2010; Kretzschmar et al., 2012; Ko et al., 2014; Zhang K. et al., 2014; Kang et al., 2015; Li et al., 2017; Zhao et al., 2019), sugars will eventually be exported transporter (SWEET) (Kanno et al., 2016), purine permease (PUP) (Qi and Xiong, 2013; Zurcher et al., 2016; Xiao et al., 2019, 2020), and Aza-guanine resistant (AZG) (Tessi et al., 2020). Although adequate knowledge is available on the biosynthesis and signaling of those hormones, knowledge regarding their transport is limited. More transporters must be identified for a comprehensive understanding of the transport mechanism of different hormones.

The substrate preference of a transporter must be measured for its functional characterization. Over the past few decades, the transporter activities of some transporters have been measured in heterogeneous systems by using multiple commercial isotope-labeled substrates. Auxin transporters, namely PIN1, PIN4, PIN6, PIN7, P-GLYCOPROTEIN 1 (PGP1), PGP4, and AtABCB21, were identified as auxin carriers by IAA isotope tracers ^3^H-IAA in *Arabidopsis* culture cells, yeast (*Saccharomyces cerevis*iae), BY-2 cells (*Nicotiana tabacum* L. cv. Bright Yellow 2), protoplasts, or HeLa cells (Terasaka et al., 2005; Bouchard et al., 2006; Petrasek et al., 2006; Kamimoto et al., 2012). ^14^C isotope-labeled *trans*-zeatin (^14^C-*t*Z) was used for an AtPUP14 transport activity assay in *Arabidopsis* protoplasts and seedlings, whereas ^3^H-*t*Z was used for an AZG2 activity assay in *Arabidopsis* calli (Zurcher et al., 2016; Tessi et al., 2020). ^3^H-ABA was used for an assay of transporter activities of ABA transporters AtABCG25, AtABCG30, AtABCG31, AtABCG40, and DTX50, by using heterogeneous expressions in Sf9 insect cells (from *Spodoptera frugiperda*), yeast, BY-2, or *Escherichia coli* (Kang et al., 2010, 2015; Kuromori et al., 2010; Zhang H. et al., 2014). Additionally, ^3^H-JA was used for a transport activity assay of the JA transporter AtABCG16 in yeast (Li et al., 2017). The transporter activity of *Petunia hybrida* pleiotropic drug resistance 1 (PDR1) was detected by ^3^H-GR24 in the root of *PDR1-*overexpression *Arabidopsis* (Kretzschmar et al., 2012). In addition to the isotope-labeled substrates, the unlabeled hormones were also used for measuring novel transporter activity in yeast, Sf9 insect cells, or a *Xenopus* oocytes system by gas chromatography–mass spectrometry (GC–MS) or LC–MS/MS. The transporter activities of NPF3, glucosinolate transporter 1 (GTR1), SWEET13, and SWEET14 were measured using GA in a *Xenopus* oocyte system, whereas the substrate was detected through LC–MS/MS (Saito et al., 2015; Kanno et al., 2016; Tal et al., 2016). Nevertheless, the transport activity assay methods are tedious and can only test a specific substrate at a time. More convenient and high throughput methods are required for the study of hormone transporters.

In the present study, we developed a novel approach for assaying phytohormone transporter activity based on the **t**obacco **s**yringe agroinfiltration and LC–MS/MS (TSAL) method. We validated the method by using three known transporters, namely AtABCG25 for ABA, AtABCG16 for JA, and AtPUP14 for CK. Additionally, we characterized an unknown transporter ZmABCG43 as a novel transporter involved in CK transport using this approach.

## Materials and Methods

### Plant Materials and Growth Condition

Tobacco (*N. benthamiana*) seeds were sown in the soil and grown in a growth chamber at 120 μmol m^–2^s^–1^ light intensity, 55% relative humidity, and a 16/8 h day/night regime at 24°C. *Arabidopsis* seeds were grown in 1/2 MS (Murashige–Skoog) medium (M519, PhytoTechnology Laboratories), 3% sucrose, and 0.3% phytogel (P8169, Sigma); placed for 3 d at 4°C; and then transferred to a growth chamber at 22°C, 100 μmol m^–2^s^–1^ light intensity, and a 16/8 h day/night regime at 22°C. After 10 days, the seedlings were transferred to the soil and grown under the same conditions.

### Plasmid Construction

*AtABCG16*, *AtABCG25*, and *ZmABCG4*3 were cloned into pCR8 (Invitrogen) using the sequence- and ligation-independent cloning (SLIC) method (Jeong et al., 2012) and then cloned into the binary vector pMDC43 (Curtis and Grossniklaus, 2003) using the Gateway LR Clonase II enzyme mix (11791-020, Invitrogen). The coding sequence of *AtPUP14* and *AtPUP14* promoters were cloned for the *AtPUP14_*pro*_:AtPUP14-GFP* vector into pCR8 using the SLIC method (Jeong et al., 2012) to generate AtPUP14-pCR8 and AtPUP14Pro-pCR8. *AtPUP14* was cloned from AtPUP14-pCR8 to pSAT6-N1GFP (Tzfira et al., 2005) through the LR reaction to produce the green fluorescent protein (GFP)-AtPUP14 vector, whereas pMDC163 was digested by *Xba*I and *Sac*I. The *GUS* gene was replaced by the *AtPUP14-GFP* coding sequence amplified by primers, AtPUP14 GFP-F and AtPUP14 GFP-R. *AtPUP14* promoter in AtPUP14Pro-pCR8 was cloned into AtPUP14-GFP-pMDC163 through the LR reaction to generate the *AtPUP14_*pro*_:AtPUP14-GFP* vector. Supplementary Table 1 lists all the primers used in the present study.

### Gene Transformation

Transient expression in the tobacco plant was performed according to the method described by Sparkes et al. (2006). Leaves of 25-DAG tobacco grown under a 16-h light/8-h dark regime were briefly transformed with *Agrobacterium* GV3101 (GV3101), which harbors a binary vector expressing *GFP-AtABCG16*, *GFP-AtABCG25*, *AtPUP14-GFP*, or *GFP-ZmABCG43*. After 3 days, the infected areas of tobacco leaves were examined and collected for immunoblotting, confocal imaging, and the hormone transport activity assay. The GFP-ZmABCG43 binary vector was transformed into *Arabidopsi*s using the floral dip method (Clough and Bent, 1998).

### Hormone Transporter Activity Assay

Leaf veins of the leaves infiltrated with *Agrobacterium*-harboring transporters were excluded. The leaves were then excised into 3 mm × 3 mm square leaf disks, and a sample of 0.1 g was weighed. The incubation buffer (5 mM MES-KOH buffer [pH 5.7]) with or without 1 mM of sodium vanadate was used to wash the samples twice. Hormone contents in the leaf disks were quantified into three or four samples for assessing the initial hormone profiling using a method described by Zhao et al. (2019). Simultaneously, three or four samples were incubated in the incubation buffer with or without 1 mM of sodium vanadate at 22°C for 0, 10, 20, 40, 60, and 90 min. Aliquots of 200 μL were collected at each time point and filtrated through a 0.22-μm filter membrane. Hormone quantification was performed using 30-μL extracts.

To perform the transporter activity assay in protoplasts, the protoplasts were prepared from infiltrated tobacco leaves with the GFP signal following a method described by Yoo et al. (2007). Subsequently, the protoplasts were washed using a W5 solution, resuspended in the W5 solution, and placed on ice for 30 min. After rewashing the cells with the W5 solution, 2–4 × 10^4^ protoplast cells were used for the hormone transport activity assay in W5 solution (1.0 mL) with or without 1 mM of sodium vanadate at 22°C. Aliquots of 200 μL were collected at 0, 5, 20, and 40 min for hormone quantification.

All the hormones were quantified using LC–MS/MS (QTRAP 5500, AB SCIEX). Hormones were separated using Exion LC (AB SCIEX) equipped with the Acquity UPLC BEH C18 column (2.1 mm × 100 mm, particle size: 1.7 μm). The column was maintained at 40°C, and the mobile phases for CK and 1-aminocyclopropane-1-carboxylic acid (ACC) were composed of water (A) and MeOH (B), and multistep linear gradient elution was used for separation: 5% B, 0–2.5 min; 5%–20% B, 2.5–3 min; 20%–50% B, 3–12.5 min; 50%–100% B, 12.5–13 min; 100% B, 13–15 min; 100%–5% B, 15–15.2 min; and 5% B, 15.2–18 min. The mobile phases for ABA, SA, JA, GA, IAA, and JA-Ile were composed of water (A) with 0.1% formic acid and MeOH (B) with 0.1% formic acid, and multistep linear gradient elution was used: 20% B, 0–1 min; 20%–100% B, 1–7 min; 100% B, 7–9 min; 100%–20% B, 9–9.3 min; and 20% B, 9.3–12 min, at a flow rate of 0.3 mL min^–1^. The optimized conditions for LC–MS/MS were as follows: curtain gas: 40 psi; ion spray voltage: 5500 V for the positive ion mode for CK, ABA, JA, ACC, GA and SA, and 5500 V for the negative ion mode for IAA and JA-Ile; turbo heater temperature: 600°C; nebulizing gas (Gas 1): 60 psi; heated gas (Gas 2): 60 psi. Data were processed using the MultiQuant software (version3.0.2, AB SCIEX). Hormones were accurately quantified through the internal standards.

### Immunoblotting and Confocal Imaging

Immunoblotting was performed using the method described by Zhang K. et al. (2014). A Zeiss LSM880 confocal microscope was used to capture all fluorescence images using the following excitation or emission settings: 488 nm/505–550 nm for GFP and 561 nm/600–660 nm for mCherry. Images were processed using the LSM image processing software (Zeiss).

### Step-by-Step Procedure

The step-by-step protocol of the TSAL method is presented in Supplementary File 1.

## Results

### Phytohormones Were Induced in the Agroinfiltrated Tobacco Leaves

**FIGURE 1:**
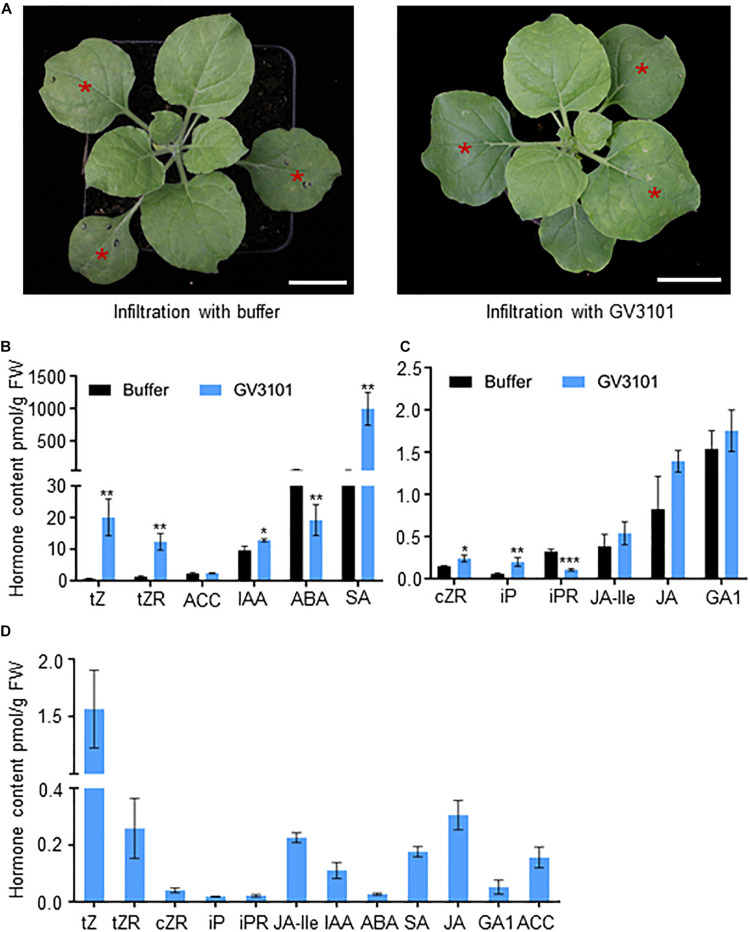
Exported hormones from tobacco leaves infiltrated with *Agrobacterium* GV3101. **(A)** Tobacco leaves 3 days after infiltration with mock and *Agrobacterium* GV3101 (GV3101) harboring free GFP. Bar = 2 cm. The leaves with a red star (*) are infiltrated leaves. **(B,C)** Hormone profiling (pmol/g fresh weight) of the tobacco leaves in **(A)**. Data are means ± SE (*n* = 3). **(D)** Quantification of hormones released from infiltrated tobacco leaves. Data are means ± SE (*n* = 5). *, **, and *** indicate *P*-values of *P* < 0.05, *P* < 0.01, and *P* < 0.001, respectively, from Student’s *t*-test.

Tobacco leaves infiltrated with *Agrobacterium* are widely used for transient protein expression assays (Sparkes et al., 2006). The concentration of several hormones was found to be significantly increased in the *Agrobacterium-*infiltrated tobacco leaves, and these hormones might be used as natural substrates for the hormone transporter activity assay. Hormones were extracted from tobacco leaves infiltrated with GV3101 harboring empty GFP vector and the mock (Figure 1A). The hormone profiling assay by LC–MS/MS exhibited detectable CKs, SA, GA, IAA, JA, JA-Ile, ABA, and ACC contents in tobacco leaves infiltrated with both the mock and GV3101. Among these, CK and SA contents were found to be increased 16-fold and 25-fold, respectively, and GA, IAA, JA, JA-Ile, and ACC contents were found to be slightly increased in tobacco leaves infiltrated with GV3101 compared with those in the mock group (Figures 1B,C). Reed et al. used the stem segment to measure auxin transporter activities (Reed et al., 2016). The present study evaluated the suitability of leaf discs for measuring transporter activities. An approach was developed using the leaf discs for the transport activity assay based on the **t**obacco **s**yringe **a**groinfiltration and **L**C–MS/MS (TSAL) method (Supplementary Figure 1). The leaf discs were incubated in the incubation buffer for 60 min to allow the movement of endogenous hormones through leaf tissues. Hormone contents in the incubation solution were quantified through LC–MS/MS. tZ, tZR, cZR, iP, iPR, JA-Ile, IAA, ABA, JA, SA, GA, and ACC were detectable at concentrations of 1.56, 0.25, 0.04, 0.18, 0.02, 0.22, 0.11, 0.03, 0.17, 0.30, 0.05, and 0.16 pmol/g fresh weight, respectively (Figure 1D), illustrating that endogenous hormones in tobacco cells or tissues can be exported to the incubation buffer through unknown transporters or diffusion.

### Detection of Hormone Transporter Activity in Leaf Discs Using the TSAL Method

**FIGURE 2:**
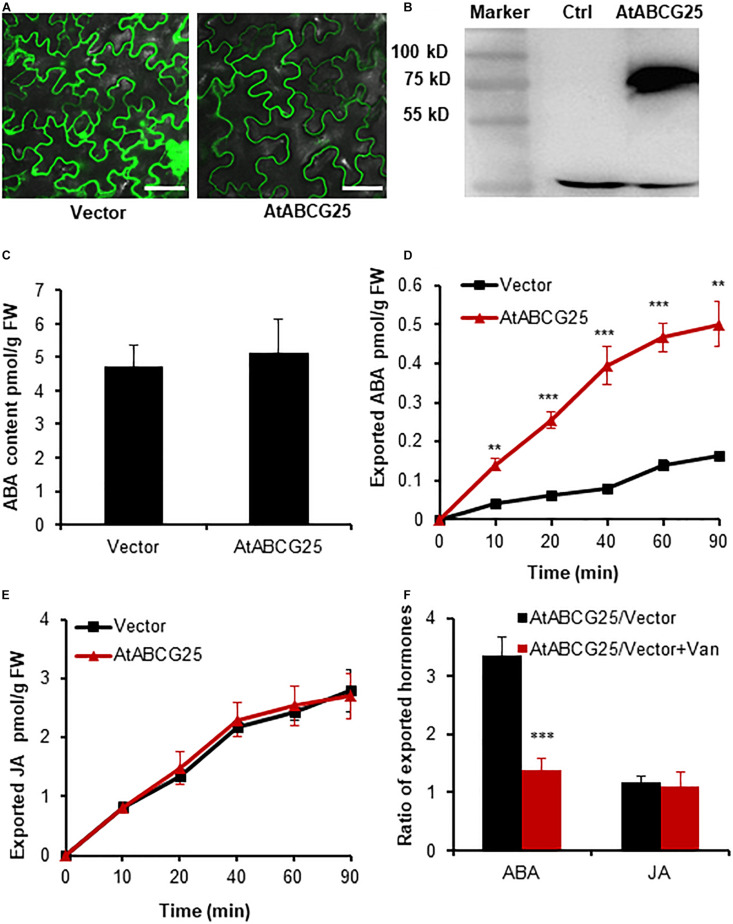
AtABCG25-mediated efflux transport of ABA in agroinfiltrated tobacco leaves. **(A)** Transient expression of *GFP* and *GFP-ABCG25* in tobacco leaves imaged by a confocal microscope. Bar = 50 μm. **(B)** Immunoblot analysis of GFP*-*AtABCG25 fusion protein expression in tobacco leaves. M, marker; G25, total protein from tobacco leaves with transient expression of *35S:GFP-AtABCG25*; Ctrl, control, total protein from non-transgenic tobacco leaves. **(C)** Quantification of ABA in tobacco leaves infiltrated with GV3101 harboring an empty vector and *GFP-ABCG25* before efflux experiment. **(D,E)** Quantification of exported ABA **(D)** and JA **(E)** from tobacco leaves transformed with *GFP-AtABCG25* and free *GFP* at the time points of 0, 10, 20, 40, 60, and 90 min. **(F)** The ratios of exported ABA and JA from tobacco leaves expressing GFP-AtABCG25 and free GFP in the presence or absence of 1 mM of sodium vanadate at 60 min. Data are means ± SE (*n* = 4). *, **, and *** indicate *P*-values of *P* < 0.05, *P* < 0.01, and *P* < 0.001, respectively, from Student’s *t*-test.

To evaluate the transporter activities, we compared the amounts of exported hormones of the *Agrobacterium*-infiltrated leaf discs harboring a transporter and an empty vector. Three known transporters, namely ABA efflux transporter AtABCG25 (Kuromori et al., 2010), JA efflux transporter AtABCG16 (Li et al., 2017), and CK influx transporter AtPUP14 (Zurcher et al., 2016), were utilized to establish a method for the transporter activity assay. To assess the activity of AtABCG25, tobacco leaves were infiltrated with GV3101 harboring binary vectors with a *35S:GFP* and *35S:GFP-AtABCG25* expression cassette. The expression and plasma membrane sublocalization of GFP-AtABCG25 were assessed through green fluorescence observation and confirmed through immunoblotting (Figures 2A,B and Supplementary Figure 2). To obtain the levels of the endogenous hormones in the materials for the TSAL assay, the initial ABA contents were found to be similar in the leaf discs expressing free GFP and GFP-AtABCG25 before the uptake assay (Figure 2C). The same batch of leaf discs was incubated in the incubation buffer for different time periods (0, 10, 20, 40, 60, and 90 min). Endogenous hormones exported to the buffer were quantified using LC–MS/MS. At each time point, the amount of ABA exported was found to be higher in tobacco leaves expressing *AtABCG25* than in the mock leaves (Figure 2D). However, the difference between JA and its control treatment was statistically non-significant (Figure 2E). Sodium vanadate, a strong inhibitor of ABC transporters, was used to inhibit AtABCG25 efflux activity to confirm whether the difference in the exported ABA was caused by AtABCG25. The exported ABA content decreased significantly with the addition of 1 mM of sodium vanadate, whereas the difference between JA and its control treatment was statistically non-significant (Figure 2F), proving that AtABCG25 is an ABA transporter.

**FIGURE 3:**
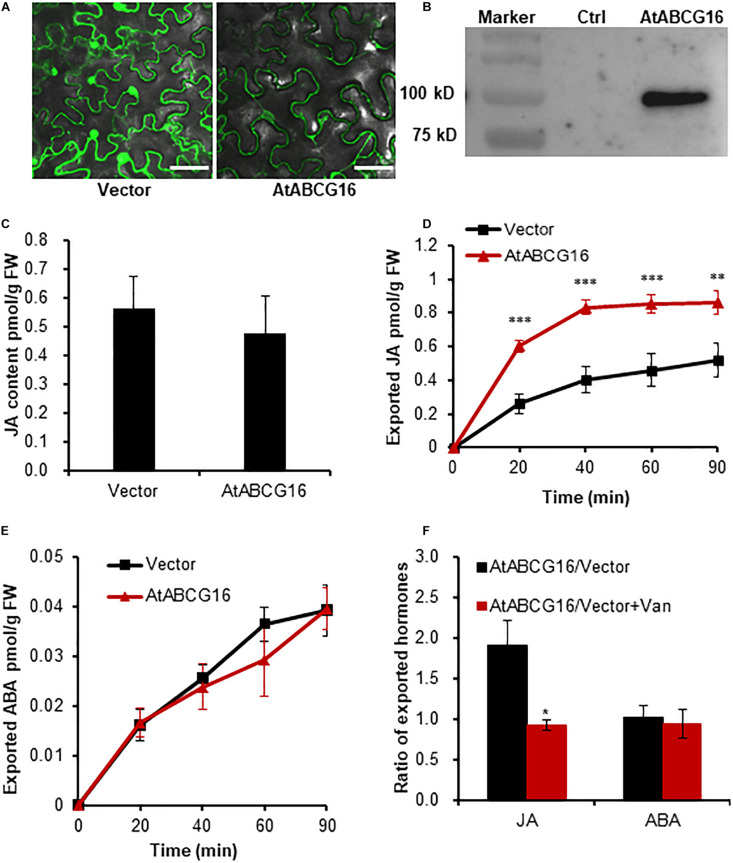
AtABCG16-mediated efflux transport of JA in agroinfiltrated tobacco leaves. **(A)** Transient expression of free *GFP* and *GFP-ABCG16* in tobacco leaves imaged by confocal microscope. Bar = 50 μm. **(B)** Immunoblot analysis of GFP-AtABCG16 fusion protein expression in tobacco leaves. M, marker; G16, total protein from tobacco leaves with transient expression of *35S:GFP-AtABCG16*; Ctrl, control, total protein from non-transgenic tobacco leaves. **(C)** Quantification of JA of tobacco leaves expressing an empty vector and *GFP-ABCG25* before efflux experiment. **(D,E)** Quantification of exported JA **(D)** and ABA **(E)** from tobacco leaves transformed with *GFP-AtABCG16* and free *GFP* at the time points of 0, 20, 40, 60, and 90 min. **(F)** The ratios of exported JA or ABA from tobacco leaves transformed with *GFP-AtABCG16* and free *GFP* in the presence or absence of 1 mM of sodium vanadate at 60 min. Data are means ± SE (*n* = 4). *, **, and *** indicate *P*-values of *P* < 0.05, *P* < 0.01, and *P* < 0.001, respectively, from Student’s *t*-test.

AtABCG16, a JA efflux transporter as reported by Li et al. (2017), was used to detect JA transport activity using the TSAL method. GFP fluorescence and immunoblotting indicated that GFP-AtABCG16 is highly expressed and properly localized on the plasma membrane of tobacco cells (Figures 3A,B and Supplementary Figure 2). The initial JA content of the leaves expressing GFP-AtABCG16 and free GFP were similar to that observed before the uptake assay (Figure 3C). After incubation in the buffer for different time periods, the leaf discs expressing GFP-AtABCG16 exported more JA but similar ABA compared with the control, supporting that AtABCG16 is a specific JA efflux transporter but not an ABA transporter (Figures 3D,E). The JA efflux transport in leaf discs expressing GFP-AtABCG16 was significantly inhibited by 1 mM of sodium vanadate (Figure 3F).

**FIGURE 4:**
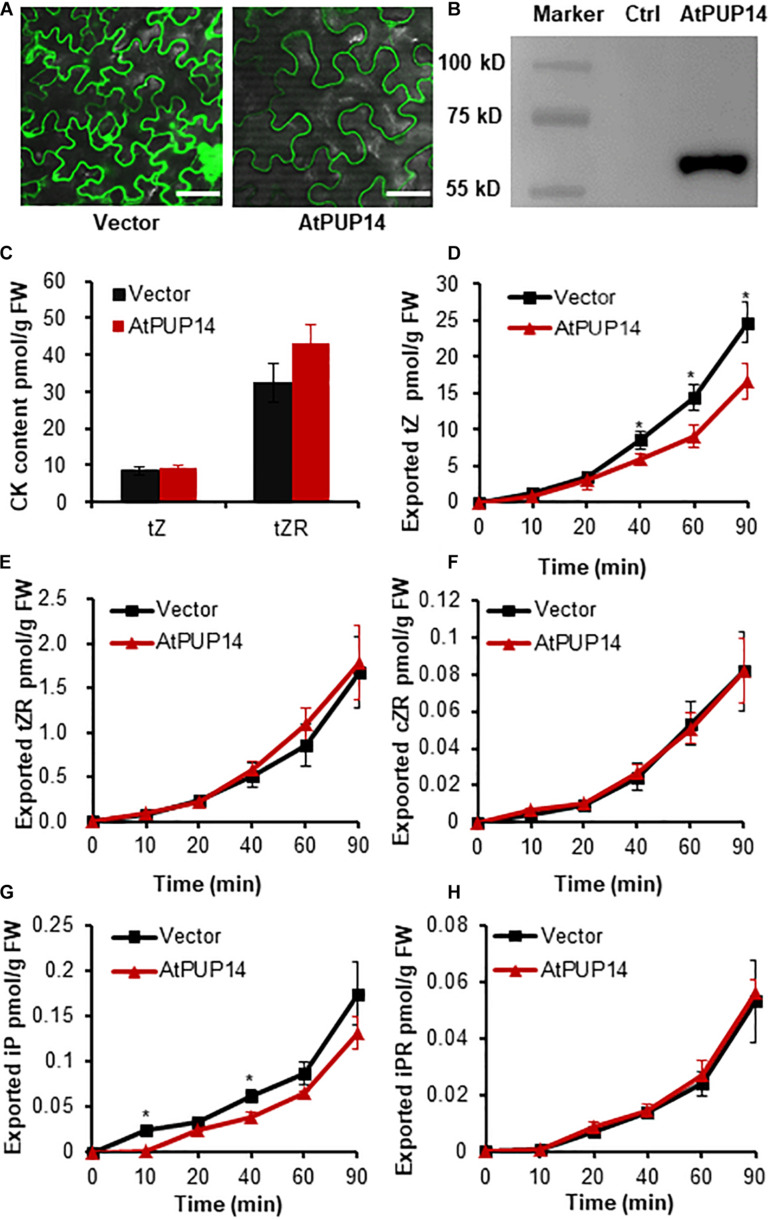
AtPUP14-mediated influx transport of cytokinins in the agroinfiltrated tobacco leaves. **(A)** Transient expression of *GFP* and *AtPUP14-GFP* in tobacco leaves imaged by confocal microscope. Bar = 50 μm. **(B)** Immunoblot analysis ofAtPUP14-GFP fusion protein expression in tobacco leaves. M, marker; AtPUP14, total protein from tobacco leaves with transient expression of *PUP14_*pro*_:AtPUP14-GFP*; Ctrl, control, total protein from non-transgenic tobacco leaves. **(C)** Quantification of tZ and tZR in tobacco leaves expressing *GFP* and *AtPUP14-GFP* before the TSAL assay. **(D–H)** Quantification of exported cytokinins of tZ, tZR, cZR, iP, and iPR from tobacco leaves transformed with *AtPUP14-GFP* or *GFP* in the incubation buffer at the time points of 0, 10, 20, 40, 60, and 90 min. Data are means ± SE (*n* = 4). *, **, and *** indicate *P*-values of *P* < 0.05, *P* < 0.01, and *P* < 0.001, respectively, from Student’s *t*-test. *t*Z, *trans*-zeatin; *t*ZR, *trans*-zeatin riboside; *c*ZR, *cis*-zeatin riboside; iP, isopentenyladenine; iPR, isopentenyladenosine.

AtPUP14, a CK influx transporter that transports free iP and tZ from apoplast to cytosol (Zurcher et al., 2016), was used for influx transporter validation. The GFP fluorescence assay and immunoblotting of free GFP and fusion protein AtPUP14-GFP confirmed the proper expression and sublocalization of AtPUP14 in the transformed leaves (Figures 4A,B and Supplementary Figure 2). Quantification of the initial contents of *trans*-zeatin (*t*Z) and *t*Z riboside (*t*ZR) in the leaf discs expressing free GFP and AtPUP14-GFP exhibited no difference (Figure 4C). After incubation for different time periods (0, 10, 20, 40, 60, and 90 min), the *t*Z and N6-(Δ2-isopentenyl) adenine (iP) exported from the AtPUP14-GFP-transformed leaf discs decreased (Figures 4D,G), whereas the difference in tZR, iP riboside (iPR), and *cis*-zeatin riboside (*c*ZR) between the AtPUP14-GFP-transformed leaf disks and control was found to be statistically non-significant (Figures 4E,F,H). The result suggests that the TSAL method is applicable for an influx transporter.

### Characterization of an Unknown Transporter ZmABCG43 Using the TSAL Method

**FIGURE 5:**
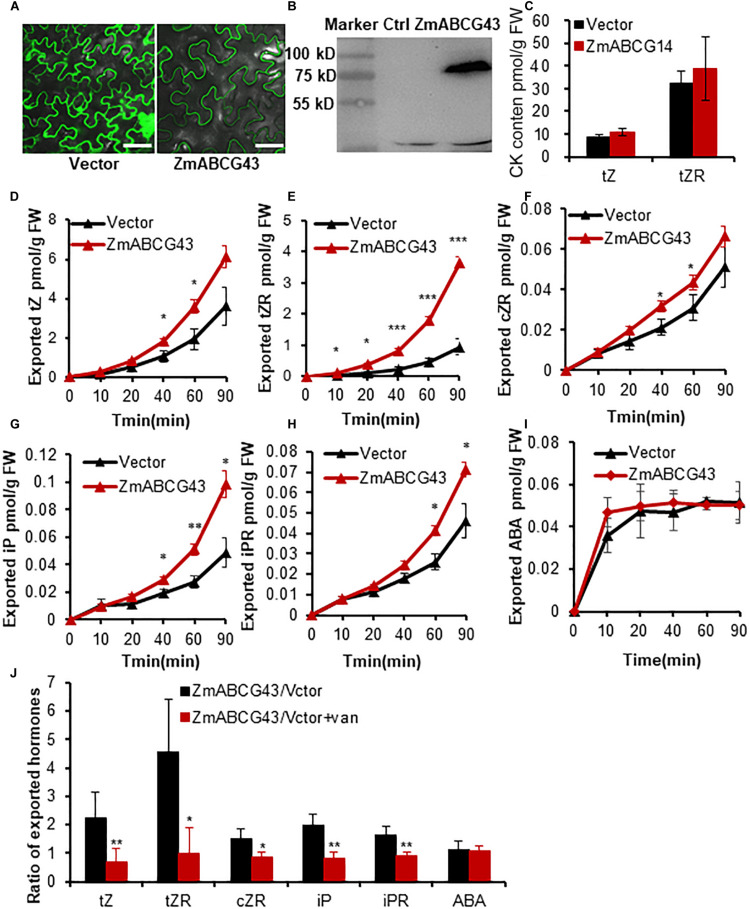
Characterization of ZmABCG43 as a transporter for cytokinins using the TSAL method. **(A)** Transient expression and sublocalization of free GFP and fusion protein GFP-ZmABCG43 in tobacco leaves. Bar = 50 μm. **(B)** Immunoblot analysis of GFP-ZmABCG43 fusion protein expression. M, marker; ZG43, total protein from tobacco leaves with transient expression of *35S:GFP-ZmABCG43*; Ctrl, control, total protein from non-transgenic tobacco leaves. **(C)** Quantification of tZ and tZR in tobacco leaves expressing GFP and GFP-*Zm*ABCG43 before the uptake assay. **(D–I)** Quantification of the exported cytokinins including tZ, tZR, cZR, cZ, iP, and iPR from tobacco leaves transformed with GFP-ZmABCG43 or GFP at the time points of 0, 10, 20, 40, 60, and 90 min. **(J)** The ratios of efflux-transported cytokinins from tobacco leaves expressing GFP-ZmABCG14 and free GFP in the presence or absence of 1 mM of sodium vanadate at 60 min. Data are means ± SE (*n* = 4). *, **, and *** indicate *P*-values of *P* < 0.05, *P* < 0.01, and *P* < 0.001, respectively, from Student’s *t*-test. ZG43, ZmABCG43.

To assess the suitability of the method for screening the substrate of a novel transporter, we detected the transport activity of an unknown transporter ZmABCG43 (an ortholog of AtABCG14) from maize (Pang et al., 2013; Supplementary Figure 3). The *ZmABCG43* coding sequence was amplified and cloned according to the website https://phytozome.jgi.doe.gov/pz/portal.html. The substrate of ZmABCG43 was screened using the TSAL method. The green fluorescence of GFP-ZmABCG43 was observed through the confocal microscope 3 days after infiltration, and the protein with a predicted size of 90 kD was detected through immunoblotting (Figures 5A,B), suggesting that the protein is strongly expressed and sublocalized in tobacco leaves. The initial contents of tZ and tZR in tobacco leaves expressing GFP-ZmABCG43 and free GFP were indistinguishable (Figure 5C). After incubation for 10, 20, 40, 60, and 90 min, the amount of exported CKs, including *t*Z, *t*ZR, *c*ZR, iP, and iPR, in the incubation buffer were significantly higher in the leaf discs expressing GFP-ZmABCG43 than in those expressing free GFP (Figures 5D–H). On the other hand, ABA, SA, JA, GA1, JA-Ile, and IAA did not exhibit any difference between *GFP-ZmABCG43* and free *GFP-*transformed leaf discs, suggesting that ZmABCG43 is an exporter specifically responsible for CKs (Figure 5I and Supplementary Figure 4). The GFP-ZmABCG43 transporter activities were appreciably inhibited in the presence of sodium vanadate (Figure 5J), confirming that ZmABCG43 is a transporter responsible for CKs.

**FIGURE 6:**
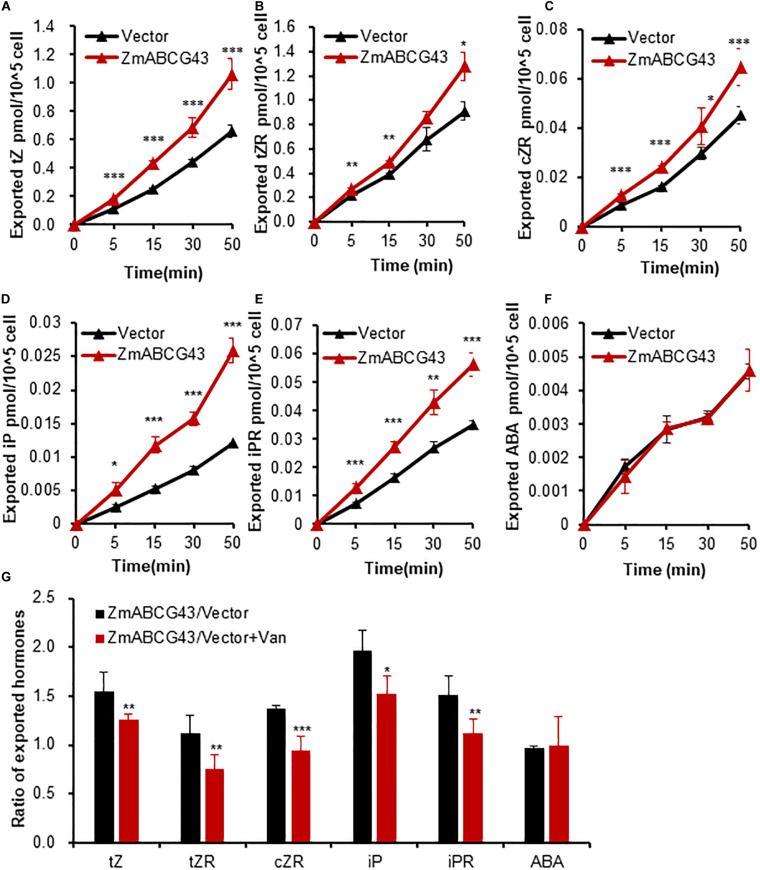
ZmABCG43-mediated efflux transport of various cytokinins in protoplasts prepared from the agroinfiltrated tobacco leaves. **(A–F)** Quantification of exported tZ, tZR, cZR, iP, and iPR from protoplasts isolated from tobacco leaves transformed with *GFP-ZmABCG43* or free *GFP* at the time points of 0, 5, 15, 30, and 50 min. **(G)** The ratios of efflux transported cytokinins from tobacco protoplasts transformed with *GFP-ZmABCG43* and free *GFP* in the presence or absence of 1 mM of sodium vanadate at 30 min. Data are means ± SE (*n* = 4). *, **, and *** indicate *P*-values of *P* < 0.05, *P* < 0.01, and *P* < 0.001, respectively, from Student’s *t*-test. ZG43, ZmABCG43.

In the transporter activity assay using leaf discs, the hormones were first exported to the leaf intercellular space and then released to the incubation buffer. A more complicated method using protoplasts was used to better evaluate the direction of the transporter ZmABCG43 at the cellular level. Protoplasts were isolated from tobacco leaves expressing GFP-ZmABCG43 and free GFP for the transport assay. The tobacco protoplasts expressing GFP-ZmABCG43 were found to export higher levels of tZ, tZR, cZR, iP, and iPR from the cells to the incubation solution than those expressing free GFP (Figures 6A–E), whereas the exported ABA levels were found to be similar (Figure 6F). Additionally, the CK efflux transport from the protoplasts expressing GFP-ZmABCG43 was significantly inhibited in the presence of sodium vanadate (Figure 6G). The consistency in results obtained using the leaf discs and protoplasts demonstrated that a transport assay using leaf discs is as reliable as that using protoplasts. Therefore, ZmABCG43 was characterized as a transporter responsible for CK efflux using the TSAL method.

### ZmABCG43 Rescued the Morphological Phenotype of *atabcg14* Mutant

**FIGURE 7:**
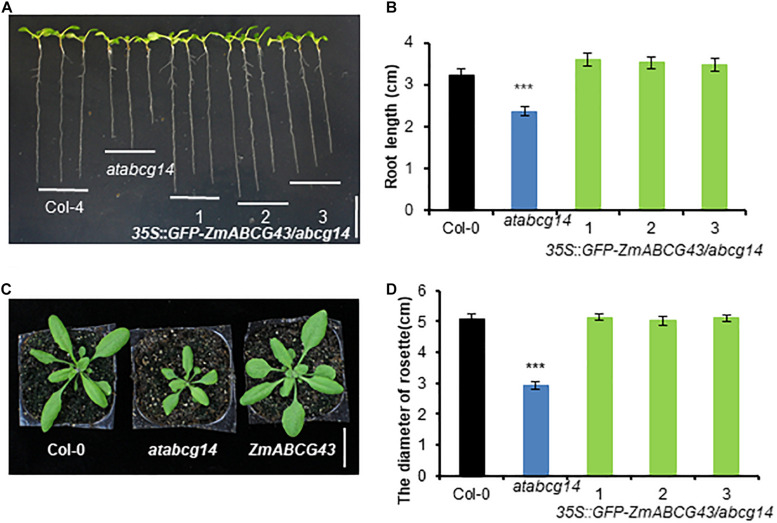
Expression of *ZmABCG43* rescued the development phenotypes of *atabcg14* mutant. **(A)** 10-DAG plants of Col-4, *atabcg14* mutant, and *atabcg14* mutant transformed with *ZmABCG43* under the CaMV35S promoter. Scale bar, 1 cm. **(B)** Root length of the seedlings in **(A)**. **(C)** 25-DAG plants of Col-4, *atabcg14* mutant, and *atabcg14* mutant transformed with *ZmABCG43* under the CaMV35S promoter. Scale bar, 2 cm. **(D)** Diameters of the rosette leaves of the plants in **(C)**. The data are means ± SD (*n* ≥ 7), ***, *P* < 0.001 (Student’s *t*-test).

To confirm the physiological function of ZmABCG43 in plants, we generated transgenic plants expressing the *ZmABCG43* gene under the control of the CaMV35S promoter in the *atabcg14* mutant, which is defective in long-distance transport of root-synthesized CKs (Ko et al., 2014; Zhang K. et al., 2014). *ZmABCG4*3 successfully complemented the growth defects of both the rosette leaf size and root length of the *atabcg14* mutant (Figures 7A–D), suggesting that *ZmABCG43* is an *AtABCG14* ortholog gene that is responsible for CK transport in maize. The *in vivo* function of ZmABCG43 is consistent with its transport activities measured using the TSAL method, suggesting that the TSAL method is suitable for the basic screening of the substrate of a novel hormone transporter.

## Discussion

*Agrobacterium-*infiltrated tobacco leaves are widely used to examine transient protein expression for multiple assays in molecular biology studies (Sparkes et al., 2006). Multiple hormones, such as CKs, SA, and IAA, were induced to high levels in the *Agrobacterium-*infiltrated tobacco leaves (Figures 1B,C). This finding is consistent with that of another study, which reported that the biosynthesis of tZ-type CKs and IAA is required for T-DNA integration to the chromosome (Morris, 1986). Farvardin et al. (2020) reported that the hormone levels in the apoplast of leaf cells are dependent on plasma membrane transporters that transfer the substrates between the cytoplasm and apoplast. In the TSAL assay, when the small leaf disks were incubated in the incubation buffer for different time periods, hormones were released from the leaf apoplast to the buffer, where they were detected through LC–MS/MS (Figure 1D). Tobacco leaves transformed with an empty vector and transporters may export varying amounts of hormones from the cytoplasm to apoplast. Thus, the transport activity can be measured by calculating the amounts of hormones in the incubation buffer.

Three known transporters, namely AtABCG25 for ABA efflux, AtABCG16 for JA efflux, and AtPUP14 for CK influx, were successfully validated using this method (Figures 2–4). These results clearly demonstrated that this method can be effectively used to assay JA, ABA, and CK transporter activity, implying that the method may be used widely for assaying the transporter activity of multiple hormones. Nevertheless, the transporter activity assays toward other hormones such as auxin, GA, BR, and SL remain to be validated. The method can also be applied for the screening of a novel transporter for a specific hormone substrate by using multiple endogenous hormones in tobacco leaves. ZmABCG43 from maize was screened *in vitro* by using this method and was confirmed *in vivo* (Figure 7). Additionally, the transporter activity assay in the protoplasts confirmed the transporter assay in the leaf discs (Figure 6), validating that the transporter activity assay using leaf discs is a rapid and reliable method.

Multiple systems have been developed to measure transporter activities by using isotope-labeled substrates in yeast (Petrasek et al., 2006), insects (Kuromori et al., 2010), *Xenopus* oocytes (Saito et al., 2015), protoplasts (Serrano et al., 2013), or plasma membrane vesicles (Zurcher et al., 2016). The TSAL method has three advantages over these methods. First, the endogenous substrates in the infiltrated tobacco leaves are more conveniently available than the isotope-labeled substrates, which are expensive or unavailable. As long as the hormone metabolites, including the active hormones or their precursors, catabolites, and conjugates, can be detected by LC–MS/MS (Pan and Wang, 2009; Wang et al., 2017; Šimura et al., 2018), their transporters can be screened from the candidate genes using the TSAL method. Second, multiple substrate screening for a transporter can be performed because the infiltrated tobacco leaves have multiple endogenous hormone substrates. Third, hormone transporters expressed in tobacco leaves can be translated and modified correctly, which can overcome the limitation of some heterogeneous expression systems.

The use of TSAL for a transporter assay or screening has some limitations. Although the GFP tag fused to the N- or C-terminal of a transporter can indicate transporter sublocalization, it occasionally affects the transporter activity. The tag-free cDNA is suggested to be constructed in parallel to express the transporters for large-scale screening. Although the method was proved to be efficient to measure the transporter that is sublocalized in the plasma membrane, an improved approach might be required to measure the activities of the transporters localized in other organelles. Additionally, the transient expression of some transporters under the CaMV35S promoter is high that may result in protein aggregation and mislocalization in tobacco leaves. A weak or native promoter may efficiently drive protein expression (Figures 4A,B; *AtPUP14-GFP* was under its native promoter). Furthermore, the exported substrates in the incubation buffer are sometimes interfered by the substrate derivatives or other cross-talk hormones. This problem can be overcome by determining background hormone levels through initial profiling of phytohormones in leaf discs before the uptake assay.

Although the TSAL method allows for the basic and rapid evaluation of multiple substrates in plants, it cannot provide sensitivity or kinetic data on phytohormone transport associated with isotope label-based methods. To obtain kinetic and more precise data, the results from the TSAL method must be further validated through classical methods using heterogeneous systems such as yeast, BY-2, *Xenopus* oocytes, and isotope-labeled substrates. The establishment of the TSAL transporter activity assay provides an approach for the basic and rapid evaluation of a substrate of a particular transporter without substrate preferences and for the large-scale screening of a novel hormone substrate-specific transporter. Moreover, the approach can be adopted for the characterization of transporters of other metabolites present in tobacco leaves that can be detected using LC–MS/MS or GC–MS.

## Data Availability Statement

The original contributions presented in the study are included in the article/Supplementary Material, further inquiries can be directed to the corresponding author/s. GenBank accession numbers are as follows: AtABCG25, AT1G71960; AtABCG16, AT3G55090; AtPUP14, AT1G19770; ZmABCG43, GRMZM2G165757.

## Author Contributions

KZ and JZ conceived and designed the experiments. JZ, MJ, JQ, MZ, and TL performed the experiments. KZ, JZ, MJ, JQ, MZ, and TL analyzed the data. JZ, MZ, and KZ wrote and revised the manuscript. All authors discussed the results and collectively edited the manuscript.

## Conflict of Interest

The authors declare that the research was conducted in the absence of any commercial or financial relationships that could be construed as a potential conflict of interest.

## Supplementary Material

Supplementary Figure 1 A flow chart of transporter activity assay by TSAL method.

Supplementary Figure 2 Colocalization of GFP-AtABC25, GFP-AtABCG16, AtPUP14-GFP, and GFP-ZmABCG43 fusion proteins with the plasma membrane marker CBL-mCherry. The fusion proteins were transiently expressed in tobacco leaf epidermal cells. Scale bars = 50 μm.

Supplementary Figure 3 Phylogenetic relationship of AtABCG14 homolog genes from *Arabidopsis* and maize. The phylogenetic tree was constructed based on a complete protein sequence alignment of half-size ABCGs from *Arabidopsis* and 12 proteins from maize with the MEGA6 software using the neighbor-joining method. AtABCG14 and GRMZM2G65757 (ZmABCG43) are highlighted in blue and red, respectively.

Supplementary Figure 4 Transport activities of ZmABCG43 toward SA, JA, GA, JA, and IAA. **(A–E)** Quantification of exported SA **(A)**, JA **(B)**, GA1 **(C)**, JA-Ile **(D)**, and IAA **(E)** from tobacco leaves transformed with *GFP-ZmABCG43* or *GFP* at the time points of 0, 10, 20, 40, 60, and 90 min.

Supplementary Table 1 Primers used in this study.
