# Supplementary figures and images for: A Tobacco Syringe Agroinfiltration-Based Method for a Phytohormone Transporter Activity Assay Using Endogenous Substrates
Source: Front Plant Sci. 2021 Apr 6;12:660966. doi: 10.3389/fpls.2021.660966 (PMC8056304; doi:10.3389/fpls.2021.660966)

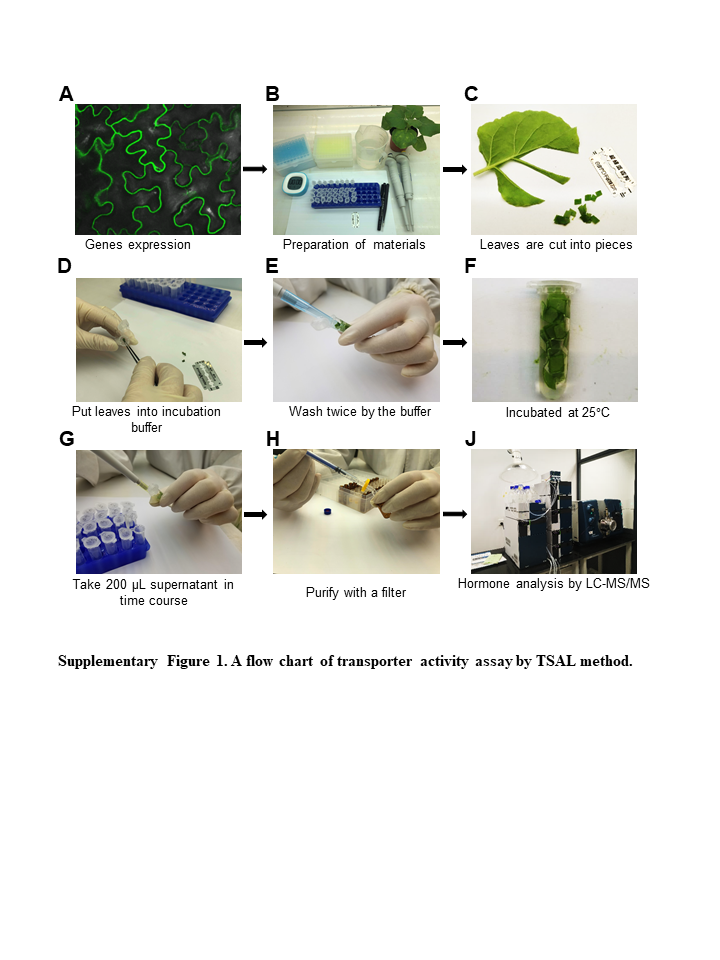

Supplement: Supplementary Figure 1 — A flow chart of transporter activity assay by TSAL method. [file Image_1.TIF]

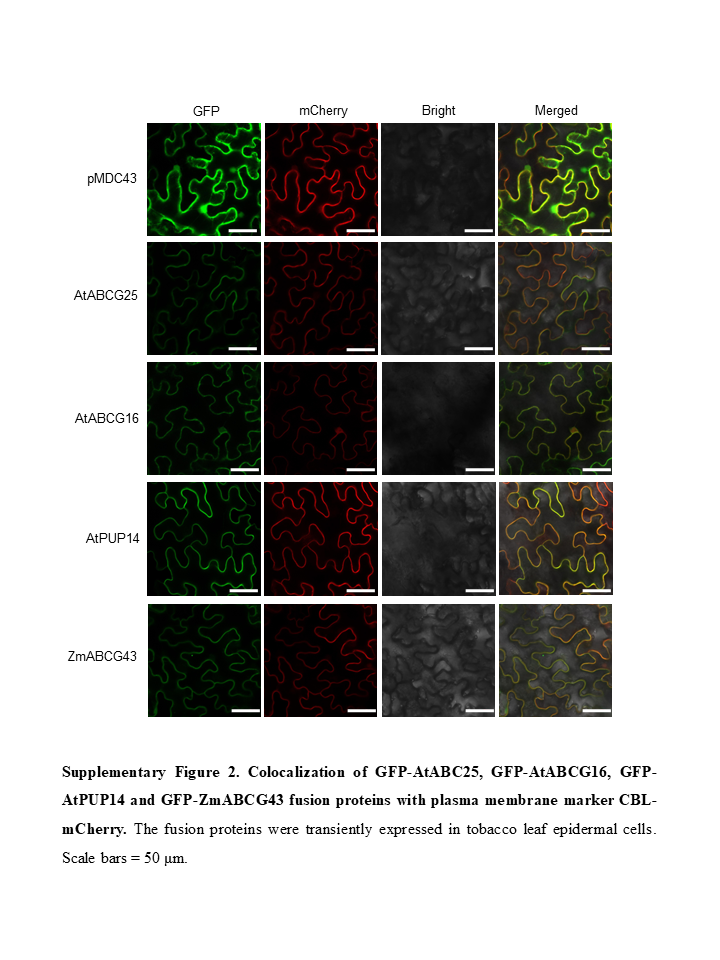

Supplement: Supplementary Figure 2 — Colocalization of GFP-AtABC25, GFP-AtABCG16, AtPUP14-GFP, and GFP-ZmABCG43 fusion proteins with the plasma membrane marker CBL-mCherry. The fusion proteins were transiently expressed in tobacco leaf epidermal cells. Scale bars = 50 μm. [file Image_2.TIF]

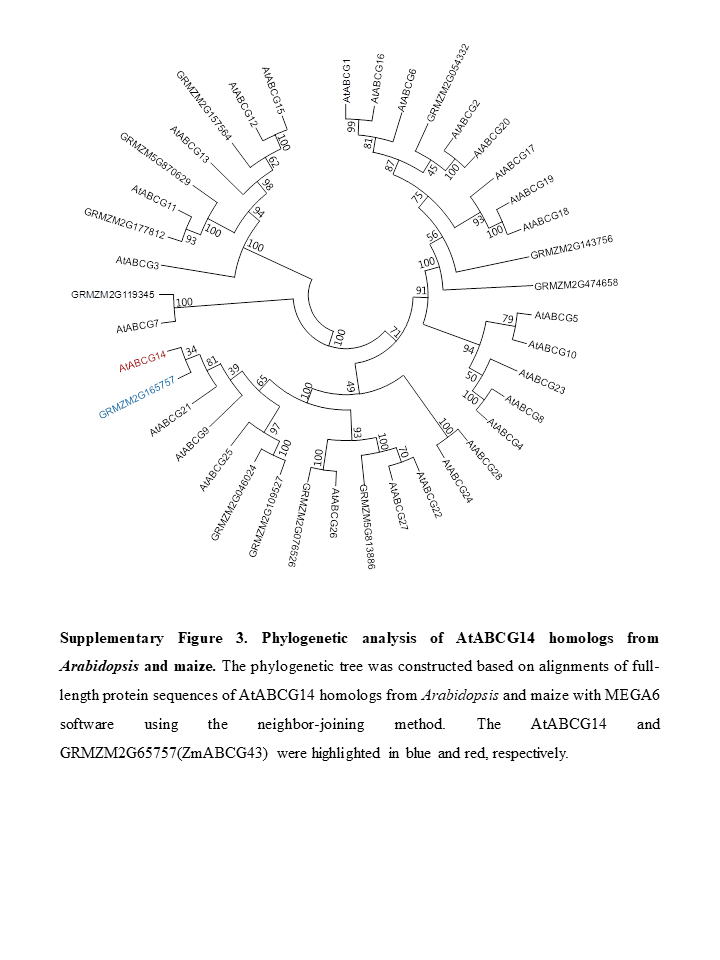

Supplement: Supplementary Figure 3 — Phylogenetic relationship of AtABCG14 homolog genes from Arabidopsis and maize. The phylogenetic tree was constructed based on a complete protein sequence alignment of half-size ABCGs from Arabidopsis and 12 proteins from maize with the MEGA6 software using the neighbor-joining method. AtABCG14 and GRMZM2G65757 (ZmABCG43) are highlighted in blue and red, respectively. [file Image_3.TIF]

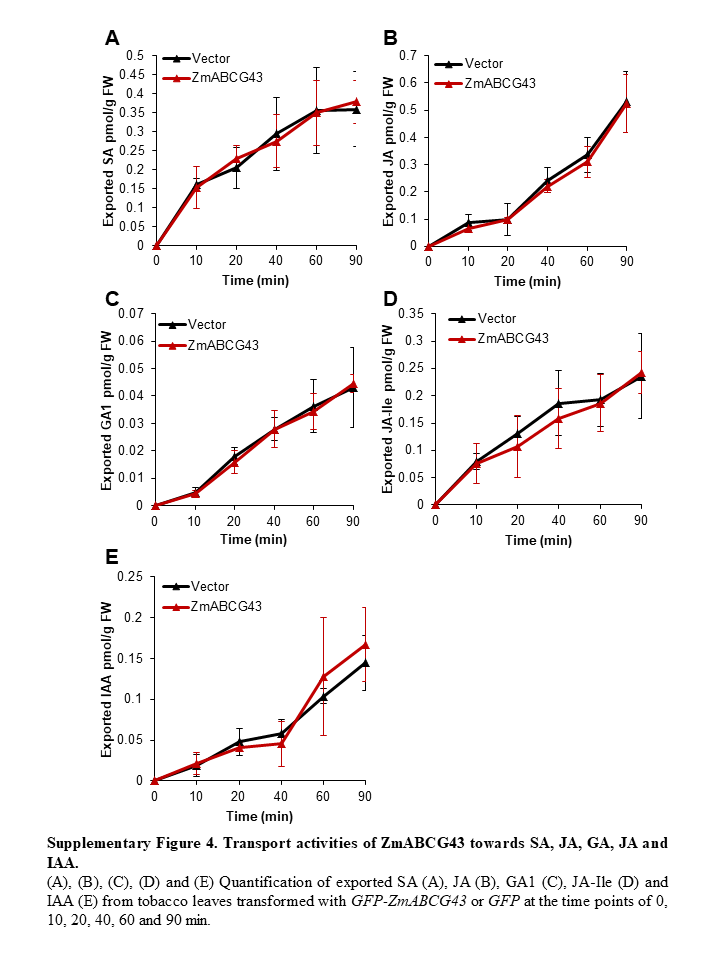

Supplement: Supplementary Figure 4 — Transport activities of ZmABCG43 toward SA, JA, GA, JA, and IAA. (A–E) Quantification of exported SA (A), JA (B), GA1 (C), JA-Ile (D), and IAA (E) from tobacco leaves transformed with GFP-ZmABCG43 or GFP at the time points of 0, 10, 20, 40, 60, and 90 min. [file Image_4.TIF]
