# Supplementary material for: A Tobacco Syringe Agroinfiltration-Based Method for a Phytohormone Transporter Activity Assay Using Endogenous Substrates
Source: Front Plant Sci. 2021 Apr 6;12:660966. doi: 10.3389/fpls.2021.660966 (PMC8056304; doi:10.3389/fpls.2021.660966)
Supplement: Supplementary Table 1 — Primers used in this study. [file Table_1.DOCX]

**Supplementary Table 1.Primers used in this study.**

| Primer name | Primer sequences (5'-3') | Restriction site underlined | Purpose |
| --- | --- | --- | --- |
| AtABCG25-F | GCAGGCTCCGAATTC ATGTCAGCTTTTGACGGCGTT |  | For AtABCG25 coding sequence |
| AtABCG25-R | AAGCTGGGTCGAATTC TTAATGTTTGATACGTCTCAAAGC |  |  |
| AtABCG16-F | GCAGGCTCCGAATTC ATGTCTCGCATACTAGTAGA |  | For AtABCG16 coding sequence |
| AtABCG16-R | AAGCTGGGTCGAATTC TCACCTCCTCTTGTTTTTGC |  |  |
| AtPUP14-F | GCAGGCTCCGAATTC ATGGCTCAGAATCAACAACC |  | For AtPUP14 coding sequence |
| AtPUP14-R | AAGCTGGGTCGAATTC CTAATAAGCCATACGATTGT |  |  |
| ZmABCG43-F | GCAGGCTCCGAATTC ATGCCGCCTGAGCTGGAGCA |  | For ZmABCG43 coding sequence |
| ZmABCG43-R | AAGCTGGGTCGAATTC TCACCTGGGCTTGAGGCGGT |  |  |
| AtPUP14 PRO-F | GCAGGCTCCGAATTC TTTTACTAGATTGCCTCTAT |  | For AtPUP14 promoter sequence |
| AtPUP14 PRO-R | GAAAGCTGGGTCGAATTC GGATTGGAACTGATTGTCGG |  |  |
| AtPUP14 GFP-F | GCTCTAGA ATGGCTCAGAATCAACAACC | XbaI | For AtPUP14-GFP sequence |
| AtPUP14 GFP-R | CGAGCTC TTACTTGTACAGCTCGTCCA | SacI |  |
