# Supplementary material for: A Tobacco Syringe Agroinfiltration-Based Method for a Phytohormone Transporter Activity Assay Using Endogenous Substrates
Source: Front Plant Sci. 2021 Apr 6;12:660966. doi: 10.3389/fpls.2021.660966 (PMC8056304; doi:10.3389/fpls.2021.660966)
Supplement: Supplementary file 6 [file Data_Sheet_1.doc]

**Protocol for assaying phytohormone transporter activity using the TSAL method**

**1. Plasmid Construction**

The gene encoding a transporter is cloned into pCR8 (Invitrogen) (Jeong et al., 2012) and then transferred to the expression vector pMDC43, pMDC85, or pMDC32 (Curtis and Grossniklaus, 2003) using the Gateway LR Clonase II enzyme mix (11791-020, Invitrogen) following the manufacturer’s instructions.

**2. Solution**

(1) LB medium: 5 g/L yeast extract (LP0021, Oxoid, American), 10 g/L tryptone (LP0042, Oxoid, American), 10 g/L NaCl (10019318, Sinopharm, China)

(2) Kanamycin (A600286, Sangon, China): 50 mg/mL

(3) Agroinfiltration buffer: 0.01 M MgCl_2_ (M2393, Sigma, American), 0.01 M MES (ME169, Amresco, American) (pH5.7) and 100 µM acetosyringone (D134406, Sigma, American)

(4) Incubation buffer：5 mM MES-KOH solution (pH5.7)

(5) HPLC Grade Methanol (67-56-1, Ourchem, China)

(6) Enzyme solution: 1–1.5% cellulase R10 (L0012, Lablead, China), 0.2–0.4% macerozyme R10 (L0021, Lablead, China), 0.4 M mannitol (M9647, Sigma, American), 20 mM KCl (P5941, Sigma, American), 20 mM MES, pH 5.7, 10 mM CaCl_2_ (C7902, Sigma, American), 0.1% BSA (A-6793, Sigma, American)

(7) W5 solution: 154 mM NaCl, 125 mM CaCl_2_, 5 mM KCl, 2 mM MES (pH 5.7)

**3. Tobacco Growth Condition**

Tobacco (*Nicotianabenthamiana*) is sown in the soil (Professional growing mix; Sun Gro Horticulture Canada Ltd., Seba Beach, AB T0E 2B0, Canada) and grows in the growth chamber at 120 μmol m^-2^s^-1^ light intensity, 50% relative humidity and a 16 h/8 h day/night regime at 24°C. 25-DAG (days after germination) tobacco plants are used for gene transformation.

**4. Procedures for uptake assay using leaf pieces**

**4.1 Tobacco transformation: TIMING up to 2 d for 4 genes**

**Day 1–2:**

Tobacco transient expression is performed as previously described (Sparkes *et al.*, 2006).

1) *Agrobacterium* GV3101 (GV3101) harboring vectors and P19 grow for 24 h in LB medium (containing 50 mg/L Kanamycin).

2) GV3101 is collected and resuspended with infiltration buffer.

3) GV3101 is placed in darkness for 3 h.

4) Leaves of 25-DAG tobacco are co-transformed with GV3101 harboring a transporter and P19.

**4.2 The assay of transporter activities using leaf pieces: TIMING up to 4 h for 4 genes**

**Day 5:**

1) After 3 d, the tobacco leaves of the infected area are checked under a confocal microscopy (Zeiss LSM880) (Figure 1). The leaf pieces with GFP signal is collected under a fluorescence dissecting stereomicroscope (Zeiss SteREO Discovery. V12).

**
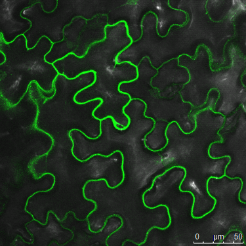
**

**Figure 1. Transient expression of a transporter in tobacco leaves**

2) The leaf blade excluding veins is cut into 3 mm*3 mm small square pieces (Figure 2A), then transferred to a 2 mL Eppendorf tubes with 1.7 mL incubation buffer (Figure 2B).


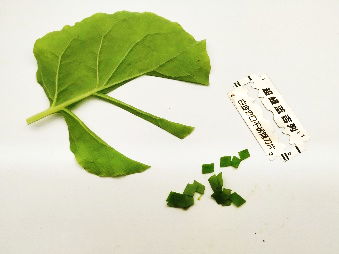

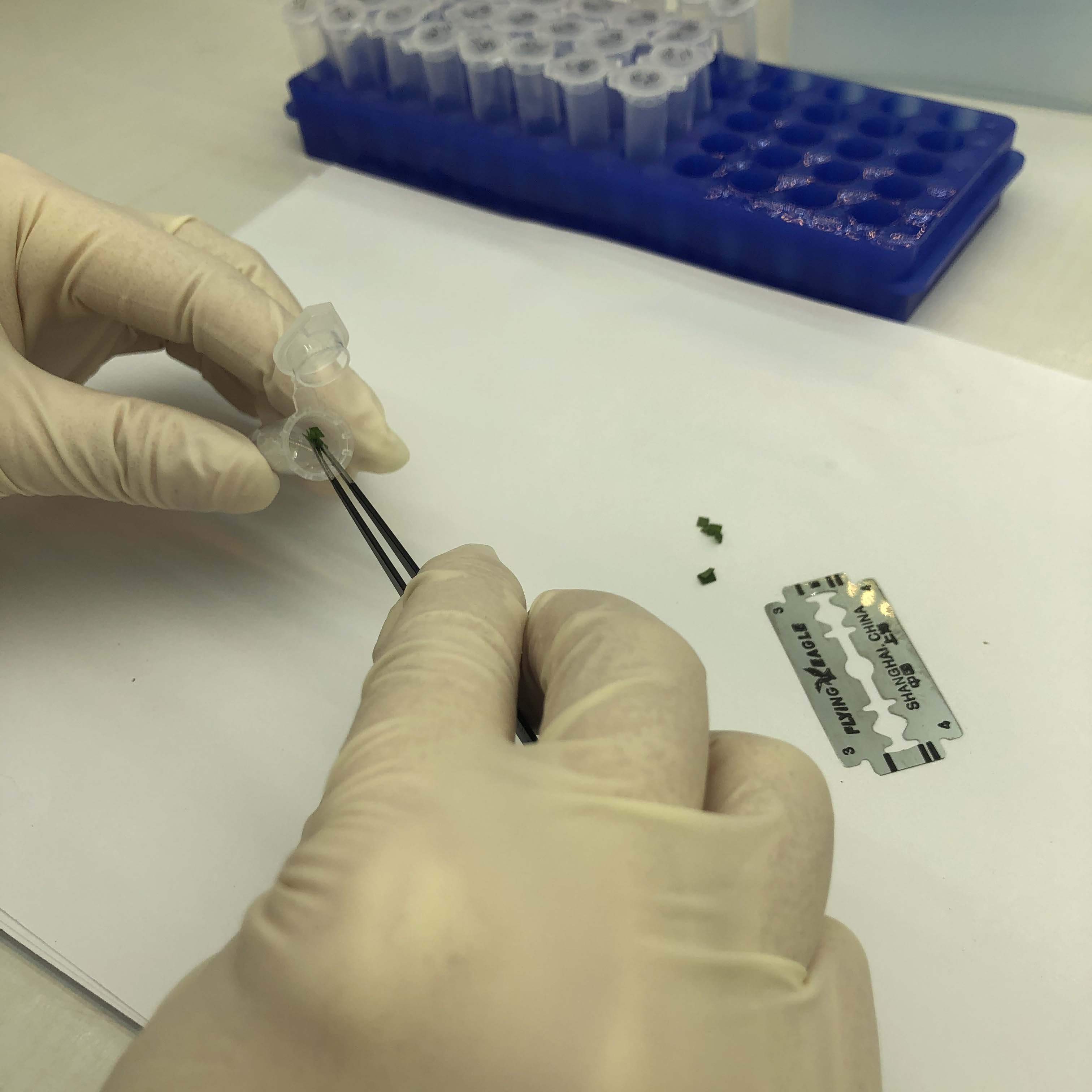


**A**

**B**

**Figure 2. Preparation of leaf pieces and the incubation**

3) The leaf pieces are washed twice with 1.5 ml incubation buffer to remove the exudates from the wound cells (Figure 3).


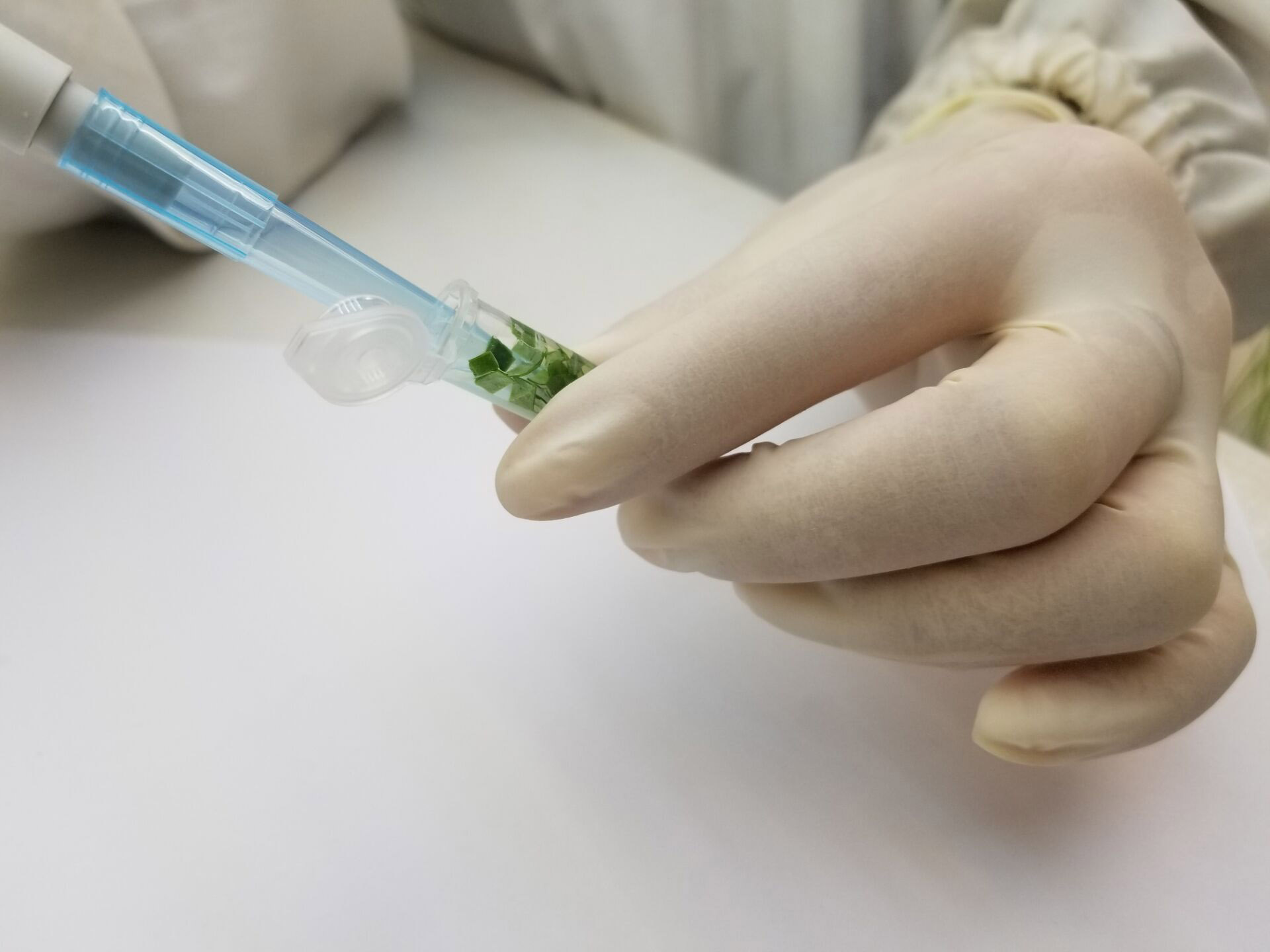


**Figure 3. Wash of the leaf pieces**

4) Add 1.7 ml incubation buffer to the tube and the sample is left on bench with gentle shaking occasionally, then 200 μL aliquots are collected at time periods of 10, 20, 40, 60, and 90 min (Figure 4).

**A**

**B**


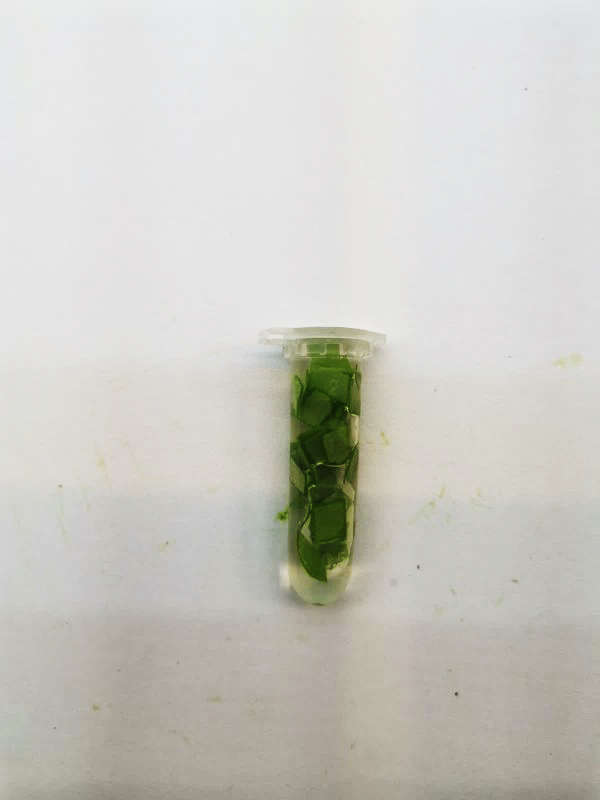

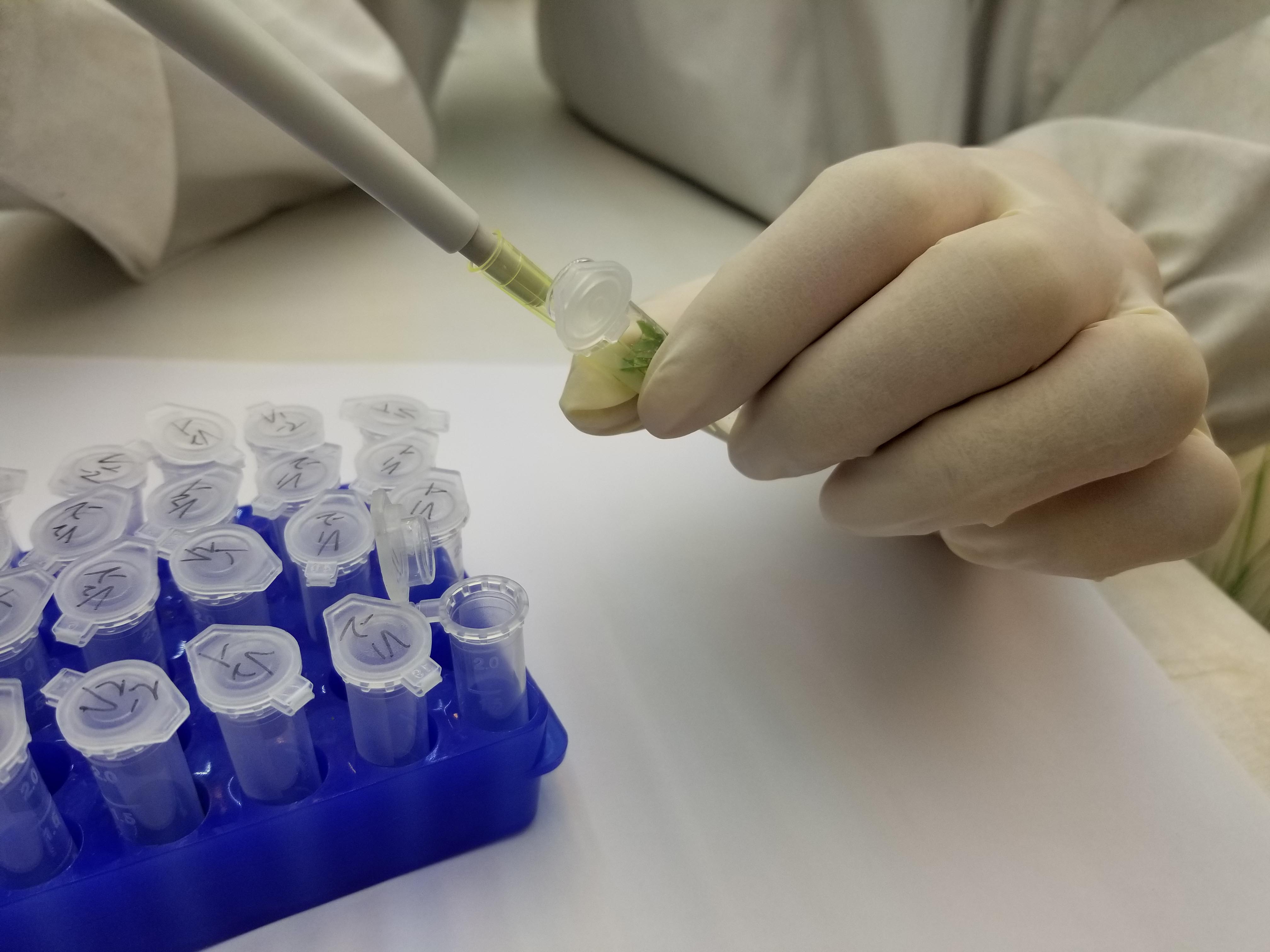


**Figure 4. Sample incubation and buffer collection**

5) The solution are filtrated with a nylon filter membrane (0.22 μm) and used for quantification by LC-MS/MS.

**Pause Point: The extracted hormones can be stored at −80°C.**

**4.3 Extraction of hormones in the leaves: TIMING up to 6 h for 4 genes**

**Day 5:**

1) 3 d after agroinfiltration, the infected areas of the tobacco leaves are harvested for hormone extraction.

**Pause Point: The harvested materials can be frozen and stored at −80°C.**

2) Leaves are grounded in liquid nitrogen, extracted with 1.6 ml extraction buffer (80% methanol with internal standards including 45 pg of ^2^H_5_-tZ, ^2^H_5_-tZR, ^2^H_6_-iP, ^2^H_6_-iPR, and 100 pg of ^2^H_6_-ABA) on a laboratory rotator for 2 h at 4°C.

3) After centrifugation (10 min, 15 000 g, 4°C), the supernatant (about 1.2 mL) is collected.

4) Another 0.6 mL extraction buffer is added to the pellet to extract the hormone and the supernatant is collected again.

5) Around 1.8 mL total supernatant is put together and dried by nitrogen gas. Then the pellet is resolved in 300 μl 30% methanol and filtrated though a filter membrane (0.22 μm).

**Pause Point: The extracted hormones can be stored at −80°C.**

**4.4 LC-MS/MS Assays: TIMING up to 30 h for 4 genes**

**Days 6–7:**

Hormones are separated by Exion LC (AB SCIEX) equipped with an Acquity UPLC BEH C18 column (2.1 mm X 100 mm, particle size of 1.7 μm). The column is maintained at 40°C and the mobile phases for cytokinin and ACC are composed of water (A) and MeOH (B) using a multistep linear gradient elution: 5% B at 0–2.5 min, 5–20% B at 2.5–3 min, 20–50% B at 3–12.5 min, 50–100% B at 12.5–13 min, 100% B at 13–15 min, 100–5% B at 15–15.2 min, and 5% B at 15.2–18 min. The mobile phases for ABA, SA, JA, GA, IAA, and JA-Ile are composed of water (A) with 0.1% formic acid and MeOH (B) with 0.1% formic acid using a multi-step linear gradient elution: 20% B at 0 to 1 min, 20 to 100% B at 1 to 7 min, 100% B at 7 to 9 min, 100 to 20% B at 9 to 9.3 min and 20% B at 9.3 to 12 min. The flow rate is 0.3 mL min^−1^.

The optimized conditions of LC-MS/MS are as follows: curtain gas, 40 psi; ion spray voltage, 5500 V for positive ion mode for cytokinins, ABA, JA, ACC, GA, and SA, 5500 V for negative ion mode for IAA, JA-Ile; turbo heater temperature, 600°C; nebulizing gas (Gas 1), 60 psi; heated gas (Gas 2), 60 psi.

The data analysis is processed by MultiQuant software (version3.0.2, AB SCIEX). Hormones are accurately quantified through the internal standards.

**5. Procedures for uptake assay using protoplasts**

**5.1 Transporter activity assay using protoplasts: TIMING up to 8 h for 4 genes**

**Day 5:**

1) The tobacco leaf area with GFP signal (shown in Figure 1) is cut under a fluorescence dissecting stereomicroscope.

The protoplasts are prepared from agroinfiltrated tobacco leaves as previously described (Yoo et al., 2007) .

2) Cut 0.5–1 mm leaf strips with fresh razor blades.

3) Put leaf strips in a Petri dish with 10 mL enzyme solution and digestion for about 3 h without shaking in the dark.

4) Filter the enzyme solution containing protoplasts with a 35–75 μm nylon mesh.

5) Spin at 100 g to pellet the protoplasts in a round-bottom tube for 2 min.

6) The protoplasts are washed once and then are resuspended in W5 solution and kept on ice for 30 min.

7) After washing one more time by W5 solution, 2–4*10^4^ protoplasts are incubated at 22°C in W5 solution (1.0 ml) with or without 1 mM sodium vanadate. Aliquots of 200 μl are collected from the incubation buffer at time points of 0, 5, 20, and 40 min for hormone quantification.

**Pause Point: The hormones in incubation solution can be stored at −80°C.**

**Day 6–7:**

8) The hormones are quantified by LC-MS/MS using the previous method (step 4.4).

**References**

Jeong, J.-Y., Yim, H.-S., Ryu, J.-Y., Lee, H.S., Lee, J.-H., Seen, D.-S., and Kang, S.G. (2012). One-Step Sequence- and Ligation-Independent Cloning as a Rapid and Versatile Cloning Method for Functional Genomics Studies. *Applied and Environmental Microbiology* 78**,** 5440-5443.

Curtis, M.D., and Grossniklaus, U. (2003). A Gateway Cloning Vector Set for High-Throughput Functional Analysis of Genes in Planta. *Plant Physiology* 133**,** 462-469.

Sparkes, I.A., Runions, J., Kearns, A., and Hawes, C. (2006). Rapid, transient expression of fluorescent fusion proteins in tobacco plants and generation of stably transformed plants. *Nat Protoc* 1**,** 2019-2025.

Yoo, S.-D., Cho, Y.-H., and Sheen, J. (2007). Arabidopsis mesophyll protoplasts: a versatile cell system for transient gene expression analysis. *Nature protocols* 2**,** 1565-1572.
